# Trap-mediated electronic transport properties of gate-tunable pentacene/MoS_2_ p-n heterojunction diodes

**DOI:** 10.1038/srep36775

**Published:** 2016-11-10

**Authors:** Jae-Keun Kim, Kyungjune Cho, Tae-Young Kim, Jinsu Pak, Jingon Jang, Younggul Song, Youngrok Kim, Barbara Yuri Choi, Seungjun Chung, Woong-Ki Hong, Takhee Lee

**Affiliations:** 1 Department of Physics and Astronomy, and Institute of Applied Physics, Seoul National University, Seoul 08826, Korea; 2 Jeonju Center, Korea Basic Science Institute, Jeonju, Jeollabuk-do 54907, Korea

## Abstract

We investigated the trap-mediated electronic transport properties of pentacene/molybdenum disulphide (MoS_2_) p-n heterojunction devices. We observed that the hybrid p-n heterojunctions were gate-tunable and were strongly affected by trap-assisted tunnelling through the van der Waals gap at the heterojunction interfaces between MoS_2_ and pentacene. The pentacene/MoS_2_ p-n heterojunction diodes had gate-tunable high ideality factor, which resulted from trap-mediated conduction nature of devices. From the temperature-variable current-voltage measurement, a space-charge-limited conduction and a variable range hopping conduction at a low temperature were suggested as the gate-tunable charge transport characteristics of these hybrid p-n heterojunctions. Our study provides a better understanding of the trap-mediated electronic transport properties in organic/2-dimensional material hybrid heterojunction devices.

Recently, two-dimensional (2D) materials have emerged as semiconductors for future nanoelectronic devices due to their ultrathin nature and favourable electronic properties^1^,^2^,^3^. Among these materials, graphene has attracted a lot of attention due to its excellent physical properties, such as high mobility, but has limits on its use as a semiconductor because of its zero band gap energy^4^,^5^,^6^. Unlike graphene, transition metal dichalcogenide (TMDC) materials, such as MoS_2_, MoSe_2_, and WSe_2_, are being largely studied as semiconductors because these materials have 2D-layered structures with sufficient band gap energy that depends on the number of stacked layers^7^,^8^,^9^. In particular, MoS_2_ has been widely studied in many device applications, such as field-effect transistors (FETs), memory, and sensors^10^,^11^,^12^. For example, it has been reported that single-layer MoS_2_-based FETs have good mobility (~tens of cm^2^/Vs) and high on/off ratios (~10^8^)^3^,^10^,^11^,^12^.

TMDCs have pristine surfaces free of dangling bonds due to van der Waals (vdW) bonding between the layers of the TMDCs, which enables vertical staking of other materials that do not have similar lattice constants to those of TMDCs. In particular, many efforts have been made to demonstrate the novel physical phenomena in vertically and laterally stacked 2D layered materials and their heterostructures^13^,^14^,^15^. Most recently, there have been a few studies on vdW heterostructures based on 2D TMDCs combined with organic materials^16^,^17^,^18^,^19^,^20^,^21^,^22^,^23^,^24^,^25^. Organic materials have several favourable features, such as flexibility, low-cost production, low-temperature processing, and a lack of dangling bonds of organic materials^26^,^27^. Previous studies demonstrated the gate-tunable electronic and optoelectronic characteristics in vdW organic/inorganic p-n hybrid heterostructures^16^,^18^,^19^,^20^,^21^,^22^,^23^,^24^,^25^. In particular, Jariwala *et al*. reported an asymmetric control over the antiambipolar characteristics in pentacene/MoS_2_ heterojunctions and observed the photovoltaic effect^16^. However, an understanding of the electrical transport properties of the organic/inorganic hybrid structures is still limited because organic materials exhibit the presence of chemical and structural defects due to imperfect crystallinity, which can often lead to charge trap densities on the order of 10^18^/cm^3^^28^. Charge trapping at the p-n heterointerface plays an important role in the performance of p-n heterojunction devices. In particular, the gate-tunable electronic properties in organic/inorganic hybrid p-n junctions are strongly affected by the charge mobility of p-type and n-type materials, which is closely related to the charge trapping phenomena^29^,^30^,^31^. Therefore, a thorough understanding of the electronic transport associated with charge trapping in hybrid heterojunctions is required to facilitate the design of electronic and optoelectronic devices based on 2D and organic semiconductors.

Here, we report trap-mediated charge transport properties in pentacene/MoS_2_ hybrid heterojunction p-n diodes. We observed that the gate-tunable electronic conduction of the p-n junction was strongly affected by trap-assisted tunnelling through the vdW gap at the heterojunction interfaces between MoS_2_ and pentacene. We also found that the energy distribution of the trap states is closely related to the carrier activation energy. Using the temperature-variable current-voltage characteristics, the gate-tunable charge transport can be explained by a space-charge-limited conduction (SCLC) and a variable range hopping (VRH) conduction, especially at low temperature.

## Results and Discussion

**Figure 1:**
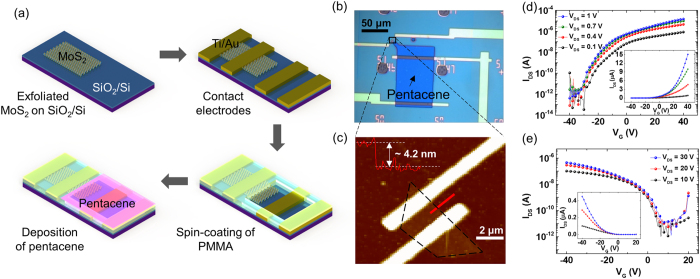
(**a**) Schematics of the device fabrication processes for the pentacene/MoS_2_ p-n heterojunction devices. (**b**) Optical image of a device. The blue area is the pentacene used in the p-type semiconductor. (**c**) AFM image of the MoS_2_ FET area. The red line shows the thickness of the MoS_2_ film (~4.2 nm). (**d**) Electrical data of a MoS_2_ FET. (**e**) Electrical data of a pentacene FET.

Figure 1(a) shows the fabrication process of the pentacene/MoS_2_ p–n junction devices. First, we transferred MoS_2_ flakes from a bulk MoS_2_ crystal (purchased from SPI Supplier, USA) onto a substrate by a mechanical exfoliation method (step 1). The substrate used in this study was a 270 nm thick SiO_2_ layer on heavily doped p^++^-Si, which is used as a common back-gate electrode. Then, we made patterns on the MoS_2_ flake and SiO_2_ to form contact electrodes using an electron beam lithography system. Au (50 nm)/Ti (5 nm) were deposited as the contact electrodes using an electron-beam evaporator (step 2). After that, we spin-coated polymethyl methacrylate (PMMA) onto the MoS_2_ surface and patterned the surface to prepare p-n junctions using the electron beam lithography system (step 3). The PMMA layer was also used as a protection layer to isolate the p-n junction area from the MoS_2_ FETs. Finally, the pentacene film (60 nm) was deposited with a thermal evaporator to fabricate the p-n heterojunctions (step 4). Here, one end of the MoS_2_ channel was in contact with the Ti/Au, and one end of the pentacene channel was in contact with Au. Note that because the work function of Ti (~4.3 eV) and Au (~5.1 eV) are close to the electron affinity of MoS_2_ (~4.2 eV)^18^,^32^ and the highest occupied molecular orbital (HOMO, ~4.9 eV)^33^ of pentacene, these metal contacts can provide good electrical contacts on MoS_2_ and pentacene. More detailed information about the fabrication process is provided in the Supplementary Information (Fig. S1). Figure 1(b,c) show an optical image of the fabricated pentacene/MoS_2_ p-n junction device and an AFM image of a MoS_2_ layer with electrodes, respectively. A MoS_2_ flake is enclosed by the black dashed line in Fig. 1(c). The red line in Fig. 1(c) shows the topological profile of the MoS_2_ flake (~4.2 nm thick), which corresponds to ~6 layers of MoS_2_.

Figure 1(d,e) show the electrical characteristics of the fabricated MoS_2_ and pentacene FET devices, respectively. MoS_2_ and pentacene exhibit n-type and p-type nature, respectively. From these figures, the field-effect mobility (*μ*) of the MoS_2_ and pentacene FETs was calculated by the following formula:


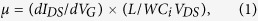


where 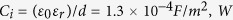 is the channel width, *L* is the channel length, *C*_*i*_ is the capacitance between the MoS_2_ or pentacene channel and the p^++^-Si gate per unit area*, ε*_*0*_ is the vacuum permittivity, *ε*_*r*_ is the dielectric constant of SiO_2,_ and d is the thickness of the SiO_2_ layer. The field-effect mobility was estimated to be ~15.2 and 0.06 cm^2^/Vs for MoS_2_ and pentacene FETs, respectively. It has been reported that band-like transport are observed in MoS_2_ devices beyond at a certain carrier density^34^. However, we could not observe such band-like transport in our MoS_2_ devices because of insufficient carrier density (See Fig. S2 in Supplementary Information). All measurements were performed in vacuum (~10^−4^ torr) to prevent unwanted effects due to moisture and oxygen from the ambient environment^35^,^36^,^37^.

**Figure 2:**
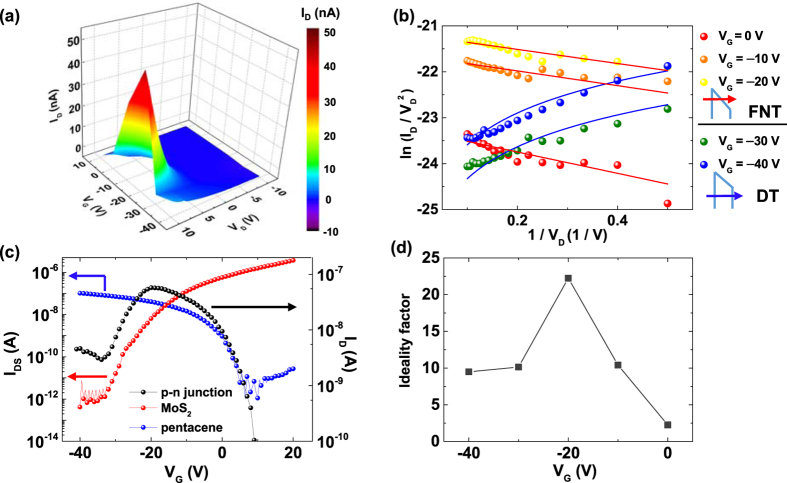
(**a**) Three-dimensional plot of the I_D_–V_D_ curves of a pentacene/MoS_2_ p–n heterojunction device with V_G_ varying from 10 V to − 40 V. (**b**) Ln (I_D_/V_D_^2^) versus 1/V_D_ plot of a pentacene/MoS_2_ p-n junction device. Schematics show the Fowler-Nordheim tunnelling (FNT) and direct tunnelling (DT) for different gate voltage conditions. (**c**) Semilogarithmic I_DS_−V_D_ curves of MoS_2_ (red) and pentacene (blue), and semilogarithmic I_D_–V_D_ curve of the pentacene/MoS_2_ p-n junction device (black). (**d**) Gate-voltage-dependent ideality factor of the pentacene/MoS_2_ p-n junction device.

Next, the gate-variable electrical characterizations were conducted for the p-n heterojunction between MoS_2_ and pentacene. Figure 2(a) shows a three-dimensional plot of the current-voltage (I_D_-V_D_) characteristics of a pentacene/MoS_2_ p-n heterojunction device at different gate voltage (V_G_) conditions. Here, the voltage was applied to the pentacene side electrode, and the MoS_2_ side electrode was grounded, while a common gate voltage was applied to both MoS_2_ and pentacene. From the I_D_-V_D_ curves, we found that the p-n heterojunction device made a transition from nearly insulating behaviour at V_G_ = 10 V to rectifying behaviour at V_G_ ≤ 0 V (see Fig. S3 in the Supplementary Information). Jariwala *et al*. have also reported similar behaviour for their pentacene/MoS_2_ heterojunctions^16^.

When heterostructure devices consist of materials that lack dangling bonds, the different materials can bond by a vdW force at the heterojunction interface^38^,^39^,^40^. Such heterostructures, such as our pentacene/MoS_2_ p-n heterojunctions, can have a vdW gap between the materials, which act as insulators. Then, the tunnelling phenomenon can occur through the a vdW gap. To analyse this tunnelling phenomenon, we used the Fowler-Nordheim plot; that is, ln(I_D_/V_D_^2^) versus 1/V_D_, as shown in Fig. 2(b). In this plot, in the Fowler-Nordheim tunnelling (FNT) regime, the charges transport through a triangular barrier, and the current is proportional to 
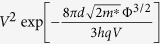
, and in the direct tunnelling (DT) regime, the carriers pass through a rectangular barrier, and the current is proportional to 
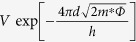
, where m* is the effective mass of the carrier, Φ is the barrier height, d is the tunnelling distance, h is Planck’s constant, q is the element charge, and V is the applied voltage^41^. Interestingly, at V_G_ = 0, −10, and −20 V, the current at a high forward bias exhibited a linear decrease in the FN plot, indicating that the transport mechanism is FNT dominant, whereas the forward current when V_G_ = −30 and −40 V exhibited logarithmic growth, indicating DT dominant transport. This type of tunnelling transformation upon changes in the gate voltage can be understood by the energy band alignment, which will be explained in a later section.

Figure 2(c) further shows the gate-tunable electrical properties for the pentacene/MoS_2_ p-n heterojunction with an asymmetric antiambipolar response. Here, the antiambipolar behaviour means that current versus gate voltage curve shows convex-up shape (black curve in Fig. 2(c)). And asymmetry is shown in such a way that the slopes of the curve on left and right side of the peak current position were different, and the current decreased more rapidly on the left side than on the right side of the current peak position. It has been reported that the asymmetric characteristics can be controlled by the ratio of the mobility, the channel length, and the series resistances of MoS_2_ and pentacene^16^. The mobility and series resistance are related by the trap density;^29^,^30^,^31^ therefore, charge traps are an important source of asymmetric transconductance of the pentacene/MoS_2_ p-n heterojunctions.

Figure 2(d) shows the ideality factors of the pentacene/MoS_2_ p–n heterojunction devices as a function of the gate voltage. The ideality factor can be extracted by the following p–n diode equation


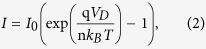


where I_0_ is the saturation current, q is the elementary charge, k_B_ is Boltzmann’s constant, T is the temperature, V_D_ is the applied voltage, and n is the ideality factor of the p-n junction. The ideality factor of our device was between 2.2 and 22.2, which is much higher than the typical value of the ideality factor (between 1 and 2) observed for conventional p-n semiconductor junctions^42^. A large ideality factor value is a common feature of vdW heterostructure devices that results from the trap state^17^,^18^,^19^. Because the ideality factor varies with the gate voltage, it suggests that the influence of the trap density on the charge transport conduction of the pentacene/MoS_2_ p-n heterojunction device also depends on the gate voltage.

**Figure 3:**
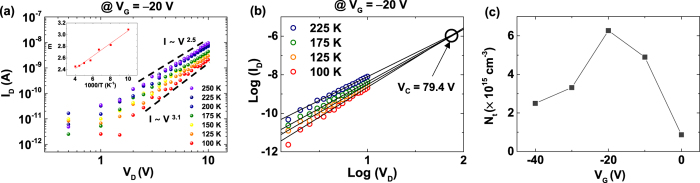
(**a**) Semilogarithmic scale log-log plot of the I_D_–V_D_ data at V_G_ = −20 V. Inset shows the exponent m in I_D_ ~ V_D_^m^ as a function of temperature. (**b**) The power-law fitting lines in (**a**) meet at a critical voltage V_C_. (**c**) The density of traps N_t_ as a function of gate voltage.

To understand the effect of trap sites in the pentacene/MoS_2_ p-n heterojunction devices, we measured the I_D_-V_D_ curves at different temperatures from 100 to 250 K with a step of 25 K. Figure 3(a) shows the log-log plot of I_D_ versus V_D_ at V_G_ = −20 V, at which the ideality factor was highest (see Fig. 2(d)). Log-log plots of I_D_ versus V_D_ at other gate voltages are provided in the Supplementary Information (Fig. S3). In Fig. 3(a), the current and voltage of the p-n heterojunction devices follow a power-law relationship of I_D_ ~V_D_^m^, in which the slope (m) varies from 2.5 to 3.1 with decreasing temperature. It is known that the power-law dependence is characteristic of SCLC with the presence of exponentially distributed charge traps^43^. SCLC means that space charges, which consist of trapped carriers near the Fermi level, make electric fields and affect carrier conduction. In SCLC with exponentially distributed traps, the current is described as


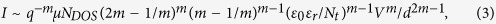


where N_t_ is the density of the trap states, *ε*_*0*_ is the vacuum permittivity, *ε*_*r*_ is the dielectric constant, μ is the mobility, N_DOS_ is the density of state in the relevant band, and d is the channel length. In SCLC with exponentially distributed traps, the power-law parameter m decreases with increasing temperature and has a larger value than 2^43^. We observed this behaviour (inset of Fig. 3(a)), which means the trap-assisted SCLC mechanism is the dominant conduction in our pentacene/MoS_2_ hybrid p-n devices. The slope of the graph (inset of Fig. 3(a)) is related to the critical temperature, which we will discuss in a later section. Also, this SCLC conduction was observed in control MoS_2_ and pentacene FETs but it is observed only in certain conditions of gate voltage and temperature (see Fig. S4 in the Supplementary Information). In the case of pentacene/MoS_2_ p-n heterojunction, the SCLC conduction was observed at all the gate voltage and temperature conditions, as shown in Fig. 3(a) and Figs S5 and S6 of the Supplementary Information. These results suggest that the SCLC conduction occurs not only in the junction of pentacene/MoS_2_, but also in the series resistance of the MoS_2_ and pentacene channels.

**Figure 4:**
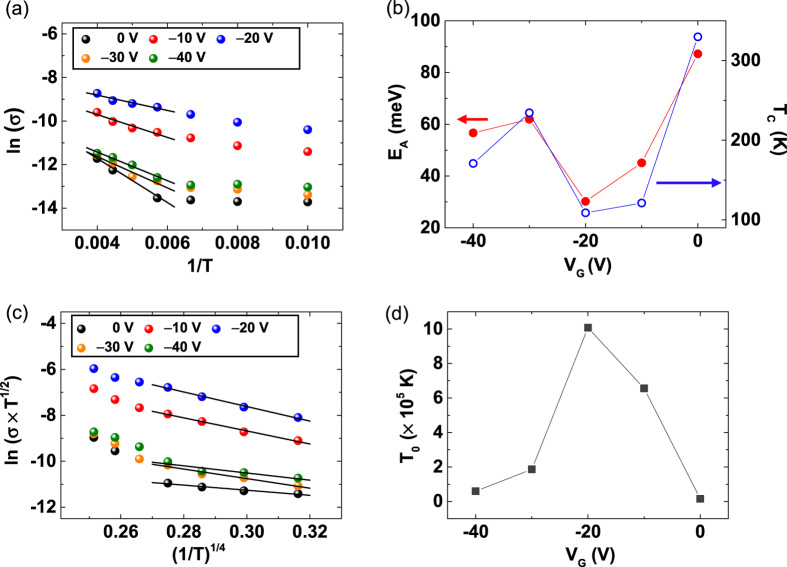
(**a**) Arrhenius plot of the conductivity σ for gate voltages from −40 V to 0 V at a fixed V_D_ = 10 V. (**b**) Activation energy (E_A_) values determined from a high-temperature region (T > ~175 K) are plotted as a function of gate voltages. The T_C_ values (a parameter indicating the energy distribution of trap sites) are also plotted. (**c**) Plot of the conductivity of pentacene/MoS_2_ hybrid p–n devices that follow the variable range hopping conduction model. (**d**) T_0_ values (a parameter showing how actively variable range hopping conduction occurs) as a function of gate voltage.

As shown in Fig. 3(b), the power-law fitted lines at different temperatures in the log-log plot meet at a critical voltage (V_C_), at which the density of traps can be calculated by the following relation: 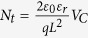, where N_t_ is the trap density in the channel, and L is the channel length^44^. As V_D_ increases, the trap sites are gradually filled by injected charge carriers from the electrode. At V_C_, the traps are completely filled, and the conductivity of the device becomes independent of temperature^44^. The V_C_ of the device was ~79.4 V at V_G_ = −20 V (Fig. 3(b)), which corresponds to N_t_ ~6.3 × 10^15^ cm^−3^. Figure 3(c) shows the density of traps N_t_ as a function of the gate voltage, as determined from the V_C_ values at different gate voltages (see Fig. S5 in the Supplementary Information). Considering that higher trap density increases the ideality factor^45^, the ideality factor variation follows the trend of trap density variation (see Figs 2(d) and 3(c)). Note that our devices had the highest current at V_G_ = −20 V, where the trap density was the largest. This is because the current also depends on the activation energy of the charge carriers at the traps, which will be explained later (Fig. 4(b)).

Figure 4(a) is an Arrhenius plot of the conductivity (σ) for gate voltages from −40 V to 0 V with a step of 10 V at a fixed V_D_ = 10 V. The activation energy (E_A_) values were determined by thermally activated transport (σ ~ exp (−E_A_/k_B_T) in a high-temperature region (T > ~175 K), and E_A_ is plotted as a function of the gate voltage, as shown in Fig. 4(b) (red filled circles). In the case of SCLC-dominant material, the larger the trap density, the larger the activation energy and the smaller the conductivity^46^. However, in our case, the tendency of the activation energy (Fig. 4(b)) does not match the tendency of the trap density (Fig. 3(c)). This tendency discrepancy can be due to the variation in the number of shallow traps that can be thermally activated in response to the gate voltage, which can be described by the critical temperature (T_C_) values as a function of the gate voltage (blue open circles), as shown in Fig. 4(b). As we mentioned previously, T_C_ can be calculated from the slope of the Arrhenius plot of m (Fig. 3(a) inset). In SCLC with exponentially distributed traps, T_C_ (a parameter that indicates how many shallow traps exist) determines the energy distribution of the trap sites; a larger T_C_ indicates a smaller number of shallow traps^46^. In Fig. 4(b), the tendency of E_A_ well matches the tendency of T_C_, which indicates that a low activation energy results in a large amount of shallow traps. Therefore, as T_C_ increases, the activation energy increases due to the decrease in the number of shallow traps^46^. And, as the activation energy increases, the conductivity decreases due to the decrease in the carrier concentration.

In contrast to the high-temperature region (T > ~175 K), in the low-temperature region, the conduction of the pentacene/MoS_2_ hybrid device does not obey thermally activated transport (see Fig 4(a,c)). The conduction in MoS_2_ and pentacene with trap sites is often explained by a VRH conduction, especially at a low temperature^47^,^48^. VRH is a conduction in which the charge carriers transport by hopping through the trap states near the Fermi level^49^. Mott suggested that the conductivity of VRH-dominant 3-dimensional (3D) materials is given by 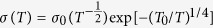, where *σ*_0_ is the characteristic conductivity, which is a function of T^−1/2^, and T_0_ is the characteristic temperature^50^. In our case, although the MoS_2_ channel has a 2D structure, we assume that the structure of our device is 3D due to the pentacene channel region which has a 3D structure. Also, we found that the 3D VRH model was the best fitting dimensional model among 1D, 2D, and 3D fittings although the VRH fittings between dimensionalities were not significantly different (see Fig. S7 in the Supplementary Information). Figure 4(c) shows that the conductivity of the pentacene/MoS_2_ hybrid p-n devices obeys the Mott’s 3D VRH model. T_0_ is the parameter showing how actively VRH conduction occurs; when T_0_ is higher, hopping occurs more actively in the conduction^47^. Figure 4(d) shows the values of T_0_ calculated from Fig. 4(c) at various gate voltages. The variation in T_0_ (Fig. 4(d)) has a similar tendency to the tendency of the variation in N_t_ (Fig. 3(c)), which indicates that as the number of trap states increases, VRH occurs more actively in the conduction. In summary, the discrepancy between the variation of the activation energy and the trap density in response to the gate voltage is due to the effect of the gate voltage-dependent number of shallow trap states, and the similar gate voltage dependency of the T_0_ and trap density indicates that the more trap density, the more active VRH is. Note that VRH conduction was observed in control MoS_2_ and pentacene at certain conditions of gate voltage and temperature (see Fig. S8 in the Supplementary Information), which suggests that VRH conduction occurs not only in the junction of pentacene/MoS_2_, but also in the series resistance of MoS_2_ and pentacene channels.

**Figure 5:**
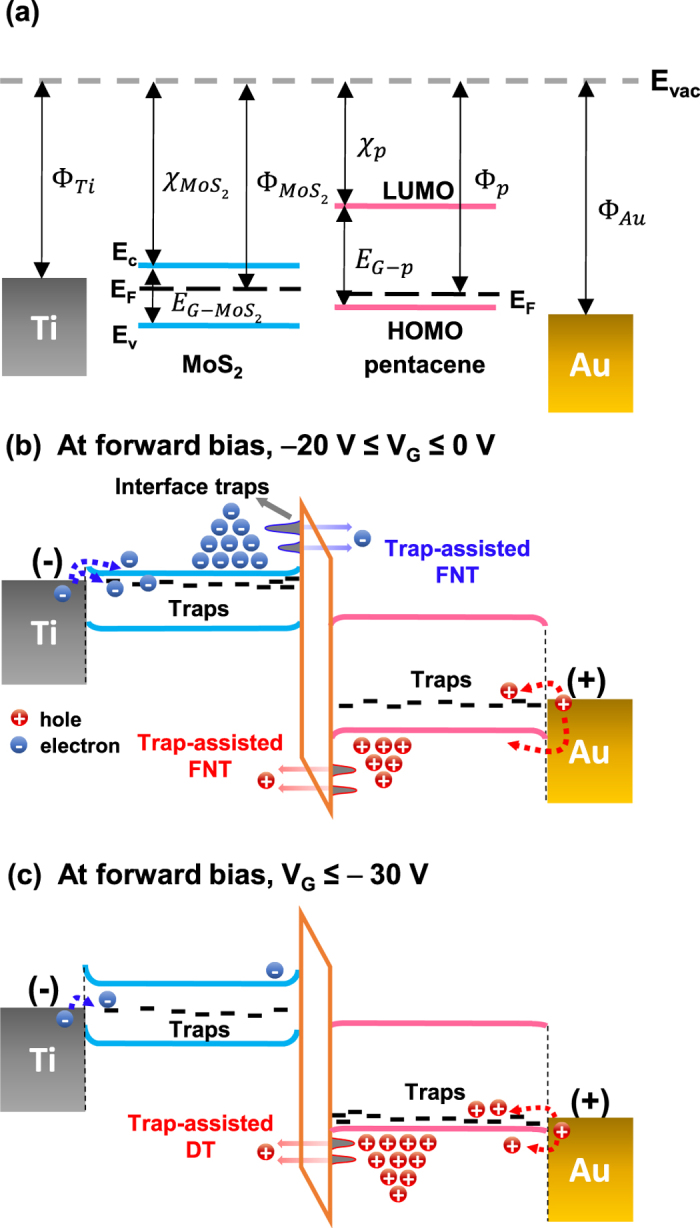
(**a**) Energy band profiles of MoS_2_ and pentacene before contacting each other. (**b,c**) Energy band alignment of the pentacene/MoS_2_ p-n junction device in the forward bias condition (**b**) at −20 V ≤ V_G_ ≤ V and (**c**) V_G_ ≤ − 30 V.

Figure 5 illustrates the energy band diagrams of the pentacene/MoS_2_ p-n heterojunction. Figure 5(a) shows the electrical parameters of the materials; the work functions (Φ) of Ti and Au are 5.1 eV and 4.3 eV, respectively; the work functions of MoS_2_ and pentacene are in the range 4.5–4.9 eV and approximately 4.5 eV, respectively; the electron affinities (χ) of MoS_2_ and pentacene are 4.0 eV and 2.7 eV, respectively; and the energy gaps (E_G_) of MoS_2_ and pentacene are 1.2 eV and 2.2 eV, respectively^32^,^33^,^51^. Figure 5(b,c) show the energy band diagram in the forward bias condition at −20 V ≤ V_G_ ≤ 0 V and V_G_ ≤ −30 V, respectively. Electrons are injected into the conduction band of MoS_2_, and holes are injected into the highest occupied molecular orbital (HOMO) of the pentacene or traps near the Fermi level. These charges tunnel through the energy barrier of the vdW gap at the pentacene/MoS_2_ junction interface. At −20 V ≤ V_G_ ≤ 0 V, both the electrons and holes can be charge carriers (Fig. 2(c)), and they pass through the triangular energy barrier via FNT (see Fig. 2(b)). It has been reported that structural defects of MoS_2_ and grain boundary of pentacene layers can act as surface charge trapping sites^52^,^53^. Also, non-uniformly deposited pentacene layer on MoS_2_ layer can contribute formation of interfacial trap sites between the layers (see Figs S11–S13 in Supplementary Information). Those traps at the interface between MoS_2_ and pentacene can assist the transport by increasing the tunnelling probability. In trap-assisted FNT, the larger the trap density, the higher the tunnelling probability^54^. And these trap density also can be affected by the gate voltage (see Fig. 3(c)). In contrast, at V_G_ ≤ −30 V, only holes can be charge carriers (Fig. 2(c)). These holes pass through the rectangular barrier via DT (see Fig. 2(b)). Similarly, the interface trap states also assist the tunnelling transport.

## Conclusions

We investigated the electrical properties of pentacene/MoS_2_ p-n heterojunction diodes at various gate voltages and temperatures. The current and conduction type of the p-n junction devices varied with the gate voltage, and the devices had a gate-bias-dependent large ideality factor. These phenomena resulted from the conduction nature of MoS_2_ and pentacene with significant trap sites. From the temperature-variable current-voltage characterization, the gate-tunable electrical characteristics of the devices were explained by a space-charge-limited conduction and a variable range hopping conduction at a low temperature. Our study helps in the understanding of the role of traps and the electrical properties of organic/2-dimensional material van der Waals heterojunction devices.

## Methods

### Fabrication of pentacene/MoS_2_ p–n heterojunction devices

First, MoS_2_ FET devices were fabricated using suitable MoS_2_ flakes that were transferred from a bulk MoS_2_ crystal by a micromechanical exfoliation method. MoS_2_ flakes were transferred to 270 nm thick SiO_2_ on a highly doped p++ Si wafer (resistivity ~5 × 10^−3^ Ω cm) that can be used as a back gate. And we identified suitable MoS_2_ flakes using an optical microscope. Measurement of the height of the MoS_2_ flakes and characterization of the surface of pentacene layer were performed by an atomic force microscope system (NX 10 AFM, Park Systems). To make patterns for the source-drain electrode, we spin-coated double electron resist layers—methyl methacrylate (MMA) (9% concentration in ethyl lactate) and polymethyl methacrylate (PMMA) (5% concentration in anisole) at 4000 rpm for each resist layer and baked the samples at 180 °C for 90 s after spin-coating each electron resist layer. The patterns for the source-drain electrode was made using an electron beam lithography system (JSM-6510, JEOL). The pattern development was performed for 30 s using a methyl isobutyl ketone/isopropyl alcohol (MIBK/IPA) (1:3) solution. Metal deposition for the source and drain electrodes was performed with an electron beam evaporator system (KVE-2004L, Korea Vacuum Tech), and the lift-off process was performed in acetone for 30 min. To make pentacene channels, PMMA was spin-coated on the sample, and pentacene was deposited using a thermal evaporator system (GVTE1000, GV-Tech).

### Device characterization

All electrical characteristics of the devices were measured using a probe station (JANIS, ST-500) with a temperature variation capability and a semiconductor parameter analyser (Keithley 4200-SCS).

## Additional Information

**How to cite this article**: Kim, J.-K. *et al*. Trap-mediated electronic transport properties of gate-tunable pentacene/MoS_2_ p-n heterojunction diodes. *Sci. Rep*. **6**, 36775; doi: 10.1038/srep36775 (2016).

**Publisher’s note**: Springer Nature remains neutral with regard to jurisdictional claims in published maps and institutional affiliations.

## Supplementary Material

Supplementary Information

## References

[b1] Novoselov K. S. . Electric field effect in atomically thin carbon films. Science 306, 666–669 (2004). 15499015 10.1126/science.1102896

[b2] Chhowalla M. . The chemistry of two-dimensional layered transition metal dichalcogenide nanosheets. Nat. Chem. 5, 263–275 (2013). 23511414 10.1038/nchem.1589

[b3] Radisavljevic B., Radenovic A., Brivio J., Giacometti V. & Kis A. Single-layer MoS_2_ transistors. Nat. Nanotechnol. 6, 147–150 (2011). 21278752 10.1038/nnano.2010.279

[b4] Li X., Wang X., Zhang L., Lee S. & Dai H. Chemically derived, ultrasmooth graphene nanoribbon semiconductors. Science 319, 1229–1232 (2008). 18218865 10.1126/science.1150878

[b5] Allen M. J., Vincent C. T. & Richard B. K. Honeycomb carbon: a review of graphene. Chemical Reviews 110, 132–145 (2009). 10.1021/cr900070d 19610631

[b6] Xu Y., Bai H., Lu G., Li C. & Shi G. Flexible graphene films via the filtration of water-soluble noncovalent functionalized graphene sheets. J. Am. Chem. Soc. 130, 5856–5857 (2008). 18399634 10.1021/ja800745y

[b7] Mak K. F., Lee C., Hone J., Shan J. & Heinz T. F. Atomically thin MoS_2_: a new direct-gap semiconductor. Phys. Rev. Lett. 105, 136805 (2010). 21230799 10.1103/PhysRevLett.105.136805

[b8] Coehoorn R. . Electronic structure of MoSe_2_, MoS_2_, and WSe_2_. I. Band-structure calculations and photoelectron spectroscopy. Phys. Rev. B. 35, 6195 (1987). 10.1103/physrevb.35.6195 9940850

[b9] Kam K. K. & Parkinson B. A. Detailed photocurrent spectroscopy of the semiconducting group VIB transition metal dichalcogenides. J. Phys. Chem. 86, 463–467 (1982).

[b10] Kim S. . High-mobility and low-power thin-film transistors based on multilayer MoS_2_ crystals. Nat. Commun. 3, 1011 (2012). 22910357 10.1038/ncomms2018

[b11] Jariwala D., Sangwan V. K., Lauhon L. J., Marks T. J. & Hersam M. C. Emerging device applications for semiconducting two-dimensional transition metal dichalcogenides. ACS Nano. 8, 1102–1120 (2014). 24476095 10.1021/nn500064s

[b12] Ganatra R. & Zhang Q. Few-layer MoS_2_: a promising layered semiconductor. ACS Nano. 8, 4074–4099 (2014). 24660756 10.1021/nn405938z

[b13] Lee C.-H. . Atomically thin p–n junctions with van der Waals heterointerfaces. Nat. Nanotechnol. 9, 676–681 (2014). 25108809 10.1038/nnano.2014.150

[b14] Kang J., Li J., Li S.-S., Xia J.- & Wang L.-W. Electronic structural moire pattern effects on MoS_2_/MoSe_2_ 2D heterostructures. Nano Lett., 13, 5485–5490 (2013). 24079953 10.1021/nl4030648

[b15] Withers F. . Light-emitting diodes by band-structure engineering in van der Waals heterostructures. Nat. Mater. 14, 301–306 (2015). 25643033 10.1038/nmat4205

[b16] Jariwala D. . Hybrid, Gate-tunable, van der Waals pn heterojunctions from pentacene and MoS_2_. Nano Lett. 16, 497–503 (2016). 26651229 10.1021/acs.nanolett.5b04141

[b17] Liu F. . Van der Waals p–n junction Based on an Organic–Inorganic Heterostructure. Adv. Funct. Mater. 25, 5865–5871 (2015).

[b18] Vélez S. . Gate-tunable diode and photovoltaic effect in an organic–2D layered material p–n junction. Nanoscale 7, 15442–15449 (2015). 26335856 10.1039/c5nr04083c

[b19] He D. . A van der Waals pn heterojunction with organic/inorganic semiconductors. Appl. Phys. Lett. 107, 183103 (2015).

[b20] Jariwala D. . Gate-tunable carbon nanotube-MoS_2_ heterojunction p-n diode. Proceedings of the National Academy of Sciences of USA 110(45), 18076–18080 (2013). 10.1073/pnas.1317226110 PMC3831469 24145425

[b21] Jariwala D. . Large-area, low-voltage, antiambipolar heterojunctions from solution-processed semiconductors. Nano. Lett. 15, 416–421 (2015). 25438195 10.1021/nl5037484

[b22] Furchi M. M., Pospischil A., Libisch F., Burgdorger J. & Muller T. Photovoltaic effect in an electrically tunable van der Waals heterojunction. Nano Lett. 14, 4785–4791 (2014). 25057817 10.1021/nl501962c PMC4138224

[b23] Yu. L. . Graphene/MoS_2_ hybrid technology for large-scale two-dimensional electronics. Nano Lett. 14, 3055–3063 (2014). 24810658 10.1021/nl404795z

[b24] Yu. L. . High-performance WSe_2_ complementary metal oxide semiconductor technology and integrated circuits. Nano Lett. 15, 4928–4934 (2015). 26192468 10.1021/acs.nanolett.5b00668

[b25] Kim. P. . Structural and electrical investigation of C_60_-graphene vertical heterostructures. ACS Nano. 9, 5922–5928 (2015). 26027690 10.1021/acsnano.5b00581

[b26] Noh Y.-Y., Zhao N., Caironi M. & Sirringhaus H. Downscaling of self-aligned, all-printed polymer thin-film transistors. Nat. Nanotechnol. 2, 784–789 (2007). 18654432 10.1038/nnano.2007.365

[b27] Cho B., Song S., Ji Y., Kim T.-W. & Lee T. Organic resistive memory devices: performance enhancement, integration, and advanced architectures. Adv. Funct. Mater. 21, 2806–2829 (2011).

[b28] Juhasz P. . Characterization of charge traps in pentacene diodes by electrical methods. Org. Electron. 17, 240–246 (2015).

[b29] Nugraha M. I. . High mobility and low density of trap states in dual‐solid‐gated PbS nanocrystal field‐effect transistors. Adv. Mater. 27, 2107–2112 (2015). 25688488 10.1002/adma.201404495

[b30] Horowitz G. & Hajlaoui M. E. Grain size dependent mobility in polycrystalline organic field-effect transistors. Synth. Met. 122, 185–189 (2001).

[b31] Salleo A. . Intrinsic hole mobility and trapping in a regioregular poly (thiophene). Phys. Rev. B 70, 115311 (2004).

[b32] Bertolazzi S., Krasnozhon D. & Kis A. Nonvolatile memory cells based on MoS_2_/graphene heterostructures. ACS Nano, 7, 3246–3252 (2013). 23510133 10.1021/nn3059136

[b33] Han W., Yoshida H., Ueno N. & Kera S. Electron affinity of pentacene thin film studied by radiation-damage free inverse photoemission spectroscopy. Appl. Phys. Lett. 103, 123303 (2013).

[b34] Jariwala D. . Band-like transport in high mobility unencapsulated single-layer MoS_2_ transistors. Appl. Phys. Lett. 102, 173107 (2013).

[b35] Park W. . Oxygen environmental and passivation effects on molybdenum disulfide field effect transistors. Nanotechnology 24, 095202 (2013). 23403849 10.1088/0957-4484/24/9/095202

[b36] Cho K. . Electric stress-induced threshold voltage instability of multilayer MoS2 field effect transistors. ACS Nano 7, 7751–7758 (2013). 23924186 10.1021/nn402348r

[b37] Wang S. D. . Contact resistance instability in pentacene thin film transistors induced by ambient gases. Appl. Phys. Lett. 94, 083309 (2009).

[b38] Padilha J. E., Fazzio A. & da Silva A. J. Van der waals heterostructure of phosphorene and graphene: Tuning the schottky barrier and doping by electrostatic gating. Phys. Rev. Lett. 114, 066803 (2015). 25723237 10.1103/PhysRevLett.114.066803

[b39] Yan R. . Esaki diodes in van der Waals heterojunctions with broken-gap energy band alignment. Nano Lett. 15, 5791–5798 (2015). 26226296 10.1021/acs.nanolett.5b01792

[b40] Lopez-Sanchez O. . Light generation and harvesting in a van der Waals heterostructure. ACS Nano 8, 3042–3048 (2014). 24601517 10.1021/nn500480u PMC3971963

[b41] Sarker B. K. & Khondaker S. I. Thermionic emission and tunneling at carbon nanotube–organic semiconductor interface. ACS Nano. 6, 4993–4999 (2012). 22559008 10.1021/nn300544v

[b42] Sze S. M. & Ng K. K. Physics of semiconductor Devices 96–98 (Wiley, 2006).

[b43] Tyagi M., Tomar M. & Gupta V. Trap assisted space charge conduction in p-NiO/n-ZnO heterojunction diode. Mater. Res. Bull. 66, 123–131 (2015).

[b44] Ghatak S. & Ghosh A. Observation of trap-assisted space charge limited conductivity in short channel MoS_2_ transistor. Appl. Phys. Lett. 103, 122103 (2013).

[b45] Giebink N. C., Wiederrecht G. P., Wasielewski M. R. & Forrest S. R. Ideal diode equation for organic heterojunctions. I. Derivation and application. Phys. Rev. B. 82, 155305 (2010).

[b46] Kumar V., Jain S. C., Kapoor A. K., Poortmans J. & Mertens R. Trap density in conducting organic semiconductors determined from temperature dependence of JV characteristics. J. Appl. Phys. 94, 1283–1285 (2003).

[b47] He G. . Conduction mechanisms in CVD-grown monolayer MoS_2_ transistors: from variable-range hopping to velocity saturation. Nano Lett. 15, 5052–5058 (2015). 26121164 10.1021/acs.nanolett.5b01159

[b48] Vissenberg M. C. J. M. & Matters M. Theory of the field-effect mobility in amorphous organic transistors. Phys. Rev. B. 57, 12964 (1998).

[b49] Germs W. C. . Charge transport in amorphous InGaZnO thin-film transistors. Phys. Rev. B 86, 155319 (2012).

[b50] Paul D. K. & Mitra S. S. Evaluation of Mott’s parameters for hopping conduction in amorphous Ge, Si, and Se-Si. Phys. Rev. Lett. 31, 1000 (1973).

[b51] Fontana M. . Electron-hole transport and photovoltaic effect in gated MoS_2_ Schottky junctions. Sci. Rep. 3, 1634 (2013). 23567328 10.1038/srep01634 PMC3620663

[b52] Addou R., Colombo L. & Wallace R. M. Surface defect on natural MoS_2_. ACS Appl. Mater. Interfaces. 7, 11921 (2015). 25980312 10.1021/acsami.5b01778

[b53] Verlaak S. & Heremans P. Molecular microelectrostatic view on electronic states near pentacene grain boundaries. Phys. Rev. B. 75, 115127 (2007).

[b54] Houng M. P., Wang Y. H. & Chang W. J. Current transport mechanism in trapped oxides: A generalized trap-assisted tunneling model. J. Appl. Phys. 86, 1488–1491 (1999).

