# Supplementary material for: Trap-mediated electronic transport properties of gate-tunable pentacene/MoS2 p-n heterojunction diodes
Source: Sci Rep. 2016 Nov 10;6:36775. doi: 10.1038/srep36775 (PMC5103186; doi:10.1038/srep36775)
Supplement: Supplementary Information [file srep36775-s1.pdf]

Supplementary Information

Trap-mediated electronic transport properties of gate-tunable pentacene/MoS_2 p–n heterojunction diodes

Jae-Keun Kim[1], Kyungjune Cho[1], Tae-Young Kim[1], Jinsu Pak[1], Jingon Jang[1], Younggul Song[1], Youngrok Kim[1], Barbara Yuri Choi[1], Seungjun Chung[1], Woong-Ki Hong*[2] & Takhee Lee*[1]

[1]*Department of Physics and Astronomy and Institute of Applied Physics, Seoul National University, Seoul 08826, Korea.*

[2]*Jeonju Center, Korea Basic Science Institute, Jeonju, Jeollabuk-do 54907, Korea.*

*Correspondence and requests for materials should be addressed to T. L. (email: tlee@snu.ac.kr) or W.-K. H. (email: wkh27@kbsi.re.kr).

1. Fabrication of pentacene/MoS_2 p–n junction device

Figure S1 shows the device fabrication processes of pentacene/MoS_2 p-n junction devices. First, SiO_2 (270 nm thick)/Si substrate was prepared and cleaned by acetone, isopropanol, and de-ionized (DI) water for 15 min each (Fig. S1(a)). Then, we transferred MoS_2 flakes from a bulk MoS_2 crystal onto the substrate by a mechanical exfoliation method (Fig. S1(b)). And we found suitable MoS_2 flakes that could be used as a field effect transistor (FET) using an optical microscope. To make patterns, we spin-coated methyl methacrylate (MMA) (9% concentration in ethyl lactate) and polymethyl methacrylate (PMMA) (5% concentration in anisole) as electron resist at 4000 rpm and baked the sample at 180°C for 90 s on a hot plate. And we made patterns on the electron resist layer using an electron beam lithography system with a 30 kV exposure. After patterning, the devices were soaked in a methyl isobutyl ketone/isopropyl alcohol (1:3) solution during 1 min for pattern development (Fig. S1(c)). Next, Au (50 nm thick)/Ti (5 nm thick) was deposited as the source and drain electrodes using an electron beam evaporator (KVE-2004L, Korea Vacuum Tech) with a deposition rate of 0.5 Å/s at pressure of ~10^{-7} torr (Fig. S1(d)). After the lift-off process with acetone, the source and drain electrode patterns were completed (Fig. S1(e)). Then, we spin-coated the PMMA on MoS_2 devices and made patterns to make p-n junction devices by using the electron lithography system (Fig. S1 (f)). PMMA coating prevents the unwanted effects that were caused by pentacene which had been deposited on MoS_2 FET channel. Finally, the pentacene active film (60 nm thick) was deposited by a thermal evaporator (GVTE1000, GV-Tech) with a deposition rate of 0.5 Å/s at pressure of ~10^{-6} torr (Fig. S1(g)).

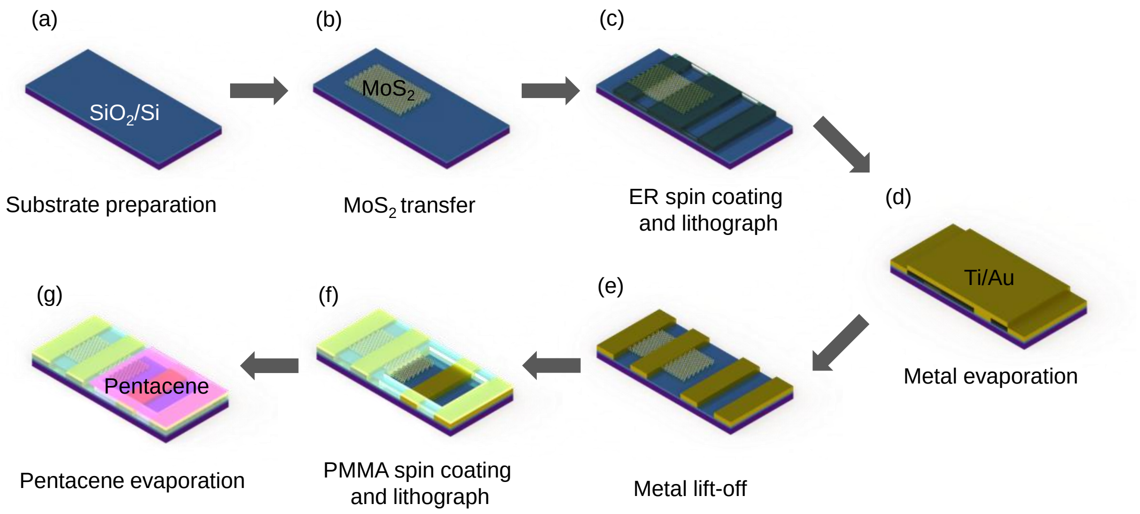

Figure S1. Schematics of fabrication process of pentacene/MoS_2 p–n junction devices.

2. Analysis of band-like transport property in MoS_2 device

Figure S2 shows the mobility versus temperature of a MoS_2 FET. If a device shows a band-like transport, mobility will have a temperature dependence such that mobility increases as temperature decreases. But in our case, the mobility of MoS_2 FET device decreased as temperature decreased, which does not support the band like transport. It is caused by insufficient carrier density of our device. Band like transport in MoS_2 FET occurr beyond a certain carrier density. The carrier density (n) of our MoS_2 FET device was estimated as ~1.63 $\times 10^{12}/cm^2$ even at a large gate voltage, $V_G = 40$ V. This value is smaller than the reported value $3.6 \times 10^{12}/cm^2$ that showed band-like transport.[S1]

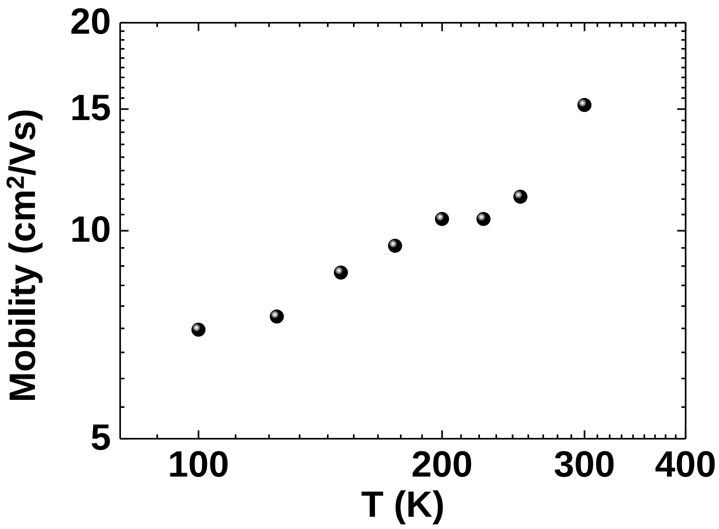

Figure S2. Semilogarithmic scale log-log plot of mobility of a MoS_2 FET versus temperature.

3. Insulating behaviour of pentacene/MoS_2 p-n junction device at V_G = 10 V

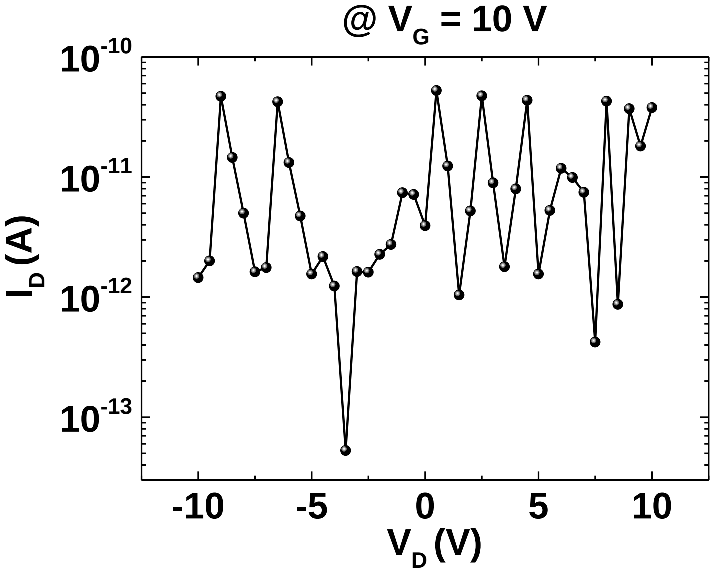

Figure S3. Insulating behaviour of pentacene/MoS_2 p-n device at V_G = 10 V.

4. Space-charge-limited conduction in MoS_2 and pentacene FETs

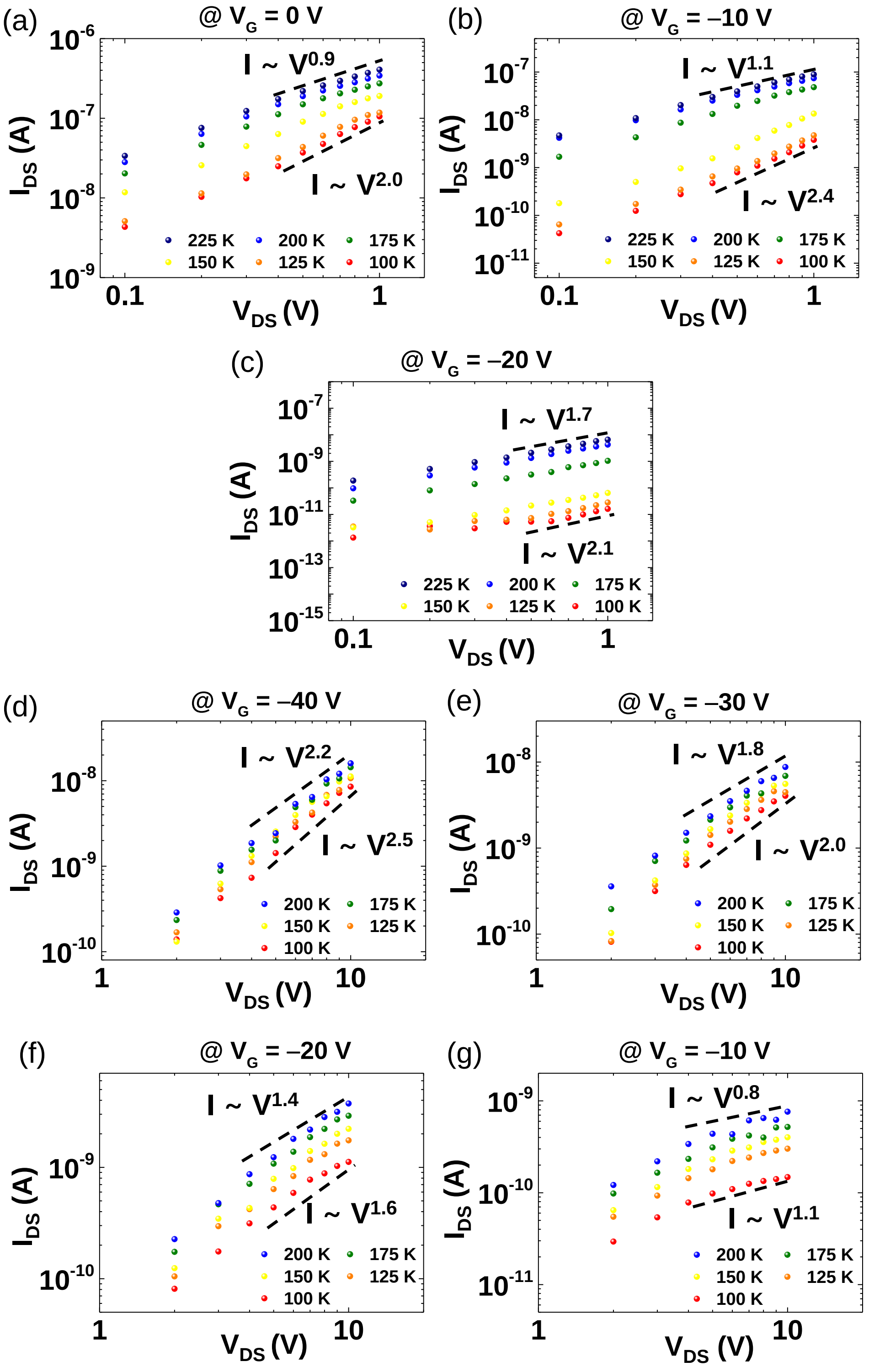

Figure S4. Semilogarithmic log-log plots of I_{DS}–V_{DS} of MoS_2 FET at V_G = (a) 0 V, (b) –10 V, and (c) –20 V and pentacene FET at V_G = (d) –40 V, (e) –30 V, (f) –20 V, and (g) –10 V.

5. Density of trap states obtained from space-charge-limited-current conduction model

Figure S5 shows the semilogarithmic scale log-log plots of I_D versus V_D of pentacene/MoS_2 p-n junction devices at V_G = (a) 0 V, (b) –10 V, (c) –30 V, and (d) –40 V. In case of space-charge-limited-current (SCLC) conduction model with exponential distribution of traps, I_D versus V_D curves at different temperatures in log-log plot meet at a critical voltage (V_C), at which the density of traps can be calculated by the following relation $N_t = 2\varepsilon_0\varepsilon_r V_C /qL^2$, where N_t is the trap density in the channel, ε_0 is the vacuum permittivity, ε_r is the dielectric constant, q is the elementary charge, and L is the channel length.[S2] We could obtain the V_C values at each gate voltage from Fig. S5. In V_G = 0 V, only I_D versus V_D curves at below 175 K intersect each other, which can be due to property of MoS_2 SCLC behavior.[S3] In MoS_2, at high temperature, the amount of free carrier density can be larger than the amount of trapped carriers. So, only I_D versus V_D curves at low temperature are appropriate in obtaining V_C value.

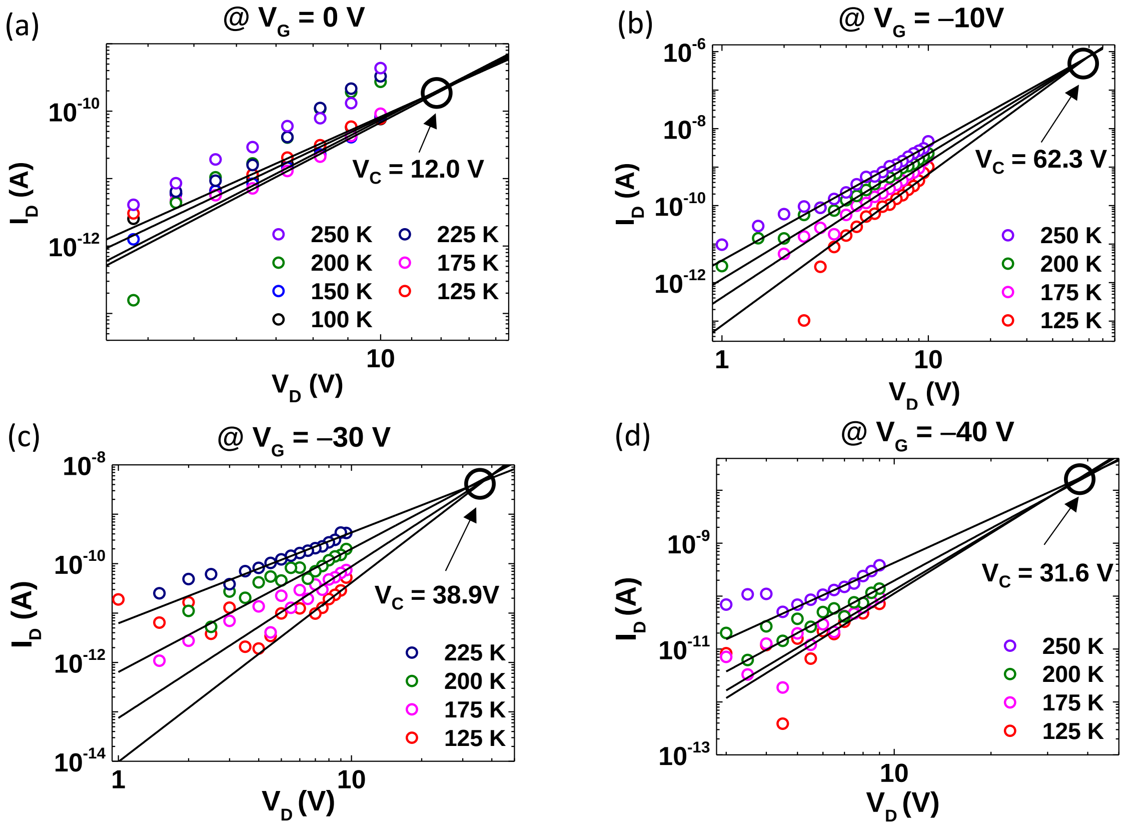

Figure S5. Semilorgarithmic scale log-log plots of the electrical characteristic curves (forward bias) of a MoS_2–pentacene p–n junction device at V_G = (a) 0 V, (b) –10 V, (c) –30 V, and (d) –40 V.

6. Power-law parameter variation of pentacene/MoS_2 p-n junction devices

Figure S6 shows power-law parameter variation of pentacene/MoS_2 at V_G = (a) 0 V, (b) –10 V, (c) –30 V, and (d) –40 V. In the SCLC with exponentially distributed traps, the power-law parameter m decreases with the increasing temperature and it is larger than two. We could confirm this SCLC property in Fig. S6.

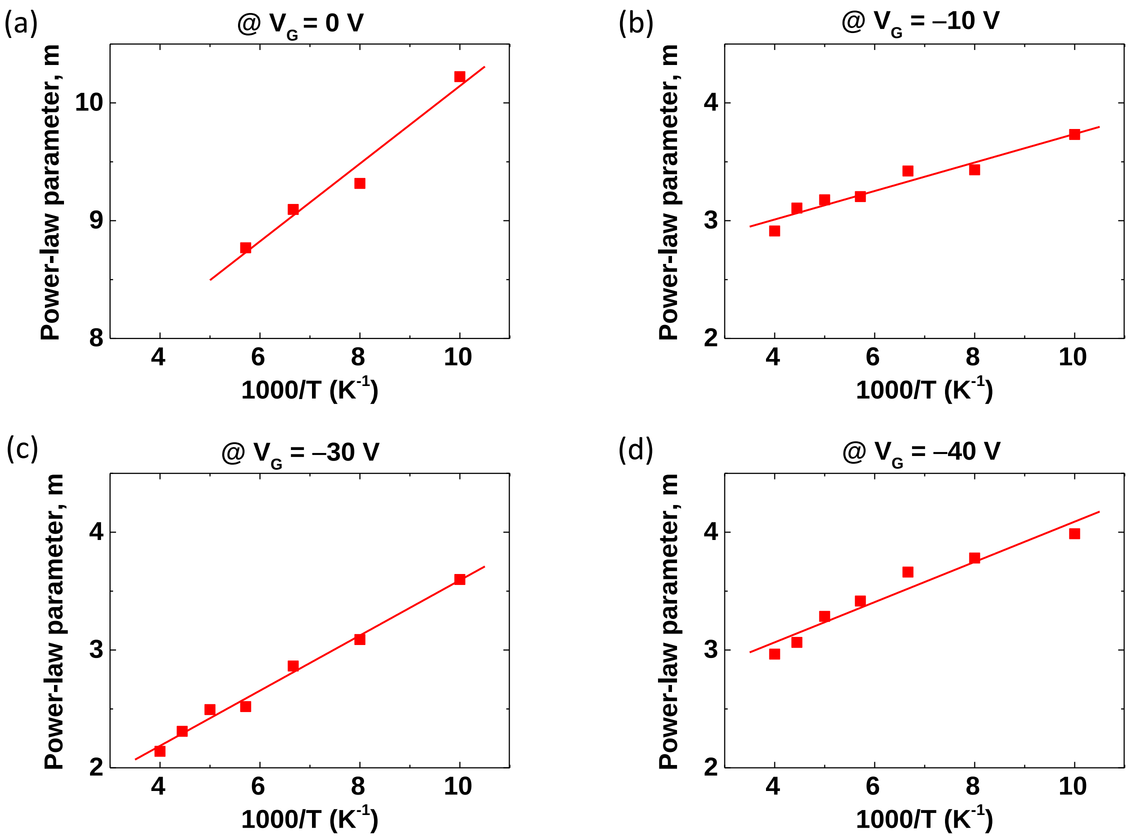

Figure S6. Arrhenius plots of power-low parameter m at V_G = (a) 0 V, (b) –10 V, (c) –30 V, and (d) –40 V.

7. Dimensional analysis of variable range hopping model in pentacene/MoS_2 p-n junction device

Figure S7 shows the VRH fittings of a pentacene/MoS_2 p-n junction device in one (1D), two (2D), and three (3D) dimension. And Table S1 shows the value of the coefficient of determination (r^2) of the fittings at each gate voltage and dimension. Here, r^2 is a parameter that describes the quality of fittings and r^2 close to 1 indicates better quality of fitting. From Table S1, the fitting values of r^2 were the most closest to 1 at 3D fitting condition although the VRH fittings between dimensionalities were not significantly different.

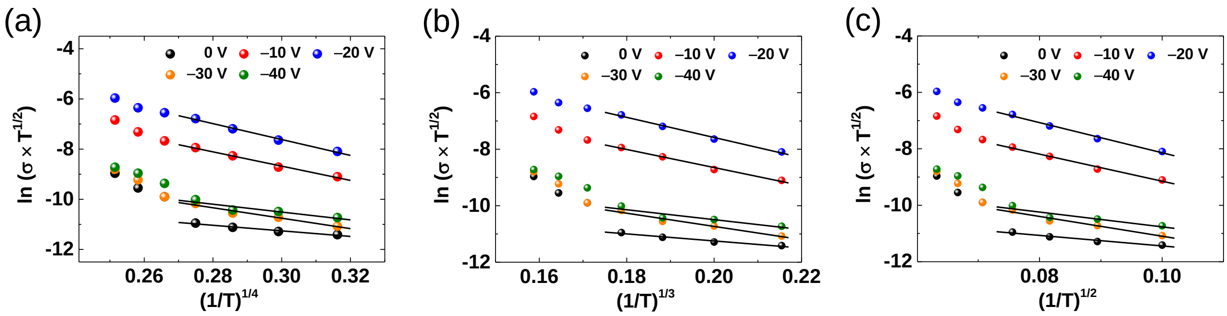

Figure S7. VRH fittings of a pentacene/MoS_2 p-n junction device in (a) 3D, (b) 2D, and (c) 1D.

Table S1. Value of r^2 of fittings at each gate voltage and dimension.

	V_G = 0 V	−10 V	−20 V	−30 V	−40 V
1D	0.948	0.981	0.984	0.944	0.803
2D	0.955	0.985	0.988	0.948	0.812
3D	0.959	0.987	0.990	0.951	0.817

8. Variable range hopping conduction in MoS_2 and petnacene FETs

Figure S8 shows the VRH fittings of control MoS_2 and pentacene FETs. For the MoS_2 FET case, VRH conduction was observed in all temperatures and all gate voltages for ON-current states. For the pentacene FET case, VRH was observed only at low temperature (T < ~175 K) and all gate voltages for ON-current states.

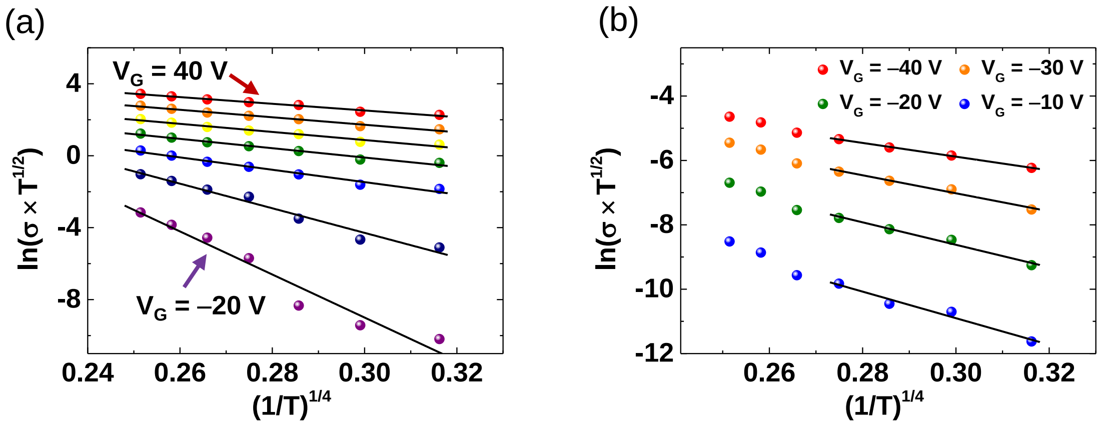

Figure S8. Three-dimensional (3D) VRH fitting of (a) MoS_2 and (b) pentacene FETs for various gate voltages with a step of 10 V.

9. Electrical characteristics of control MoS_2 and pentacene FETs with temperature variation

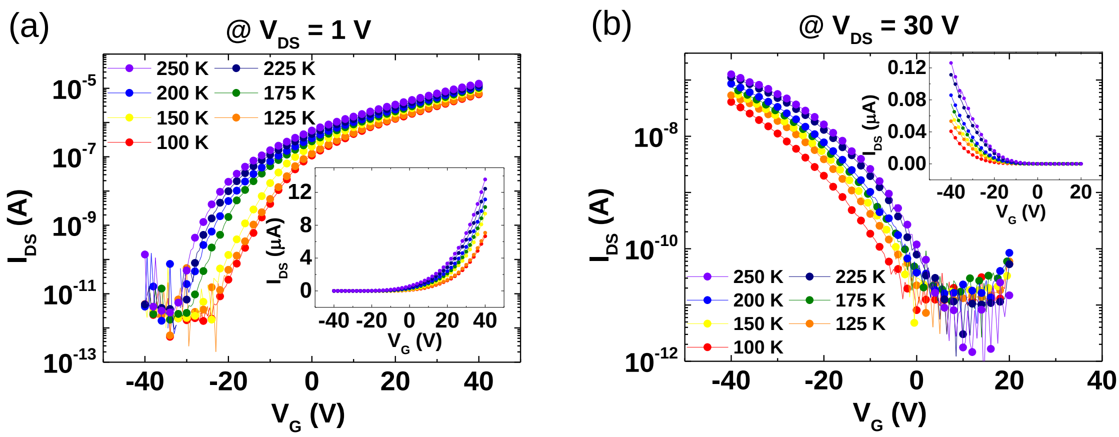

Figure S9. I_{DS}–V_G curves of (a) MoS_2 and (b) pentacene FETs at variable temperatures.

10. Schoktty barrier model analysis of control MoS_2 and pentacene FETs

Figure S10(a) shows I_{DS}–V_{DS} curves of a MoS_2 FET at various temperatures. We used the conventional thermionic emission equation for FETs data fitting to Schottky barrier model as

$$I_{DS} = AT^2 \exp\left(-\frac{q\Phi_B}{k_B T}\right)[1 - \exp(-\frac{qV_{DS}}{k_B T})]$$

In this equation, A is the Richardson constant, T is temperature, q is elementary charge, k_B is Boltzmann constant, V_{DS} is applied voltage to FET, and k_B is effective Schottky barrier height. Figure S10(b) shows the fitting result of MoS_2 FET to Schottky barrier model from the data at V_{DS} = 1 V. The high temperature data (T > ~175 K) was fitted well with Schottky barrier model (blue colored region in Figure S10(b)), whereas the low temperature data (T < ~ 175 K) wasn't fitted well with Schottky barrier model (red colored region in Figure S10(b)). At high temperature, carriers can be injected to FET by thermionic emission. However, at low temperature, effect of thermionic emission can be suppressed because of low thermal energy.

In the case of pentacene FET, Figure S10(c) shows I_{DS}–V_{DS} curves at various temperatures. And, Figure S10(d) shows the fitting result to Schottky barrier model from the data at V_{DS} = 30 V. Similar to MoS_2 FET case, high temperature data (T > ~175 K) was fitted well with Schottky barrier model (blue colored region in in Figure S10(b)), whereas the low temperature data (T < ~ 175 K) wasn't not fitted well (red colored region in Figure S10(b)).

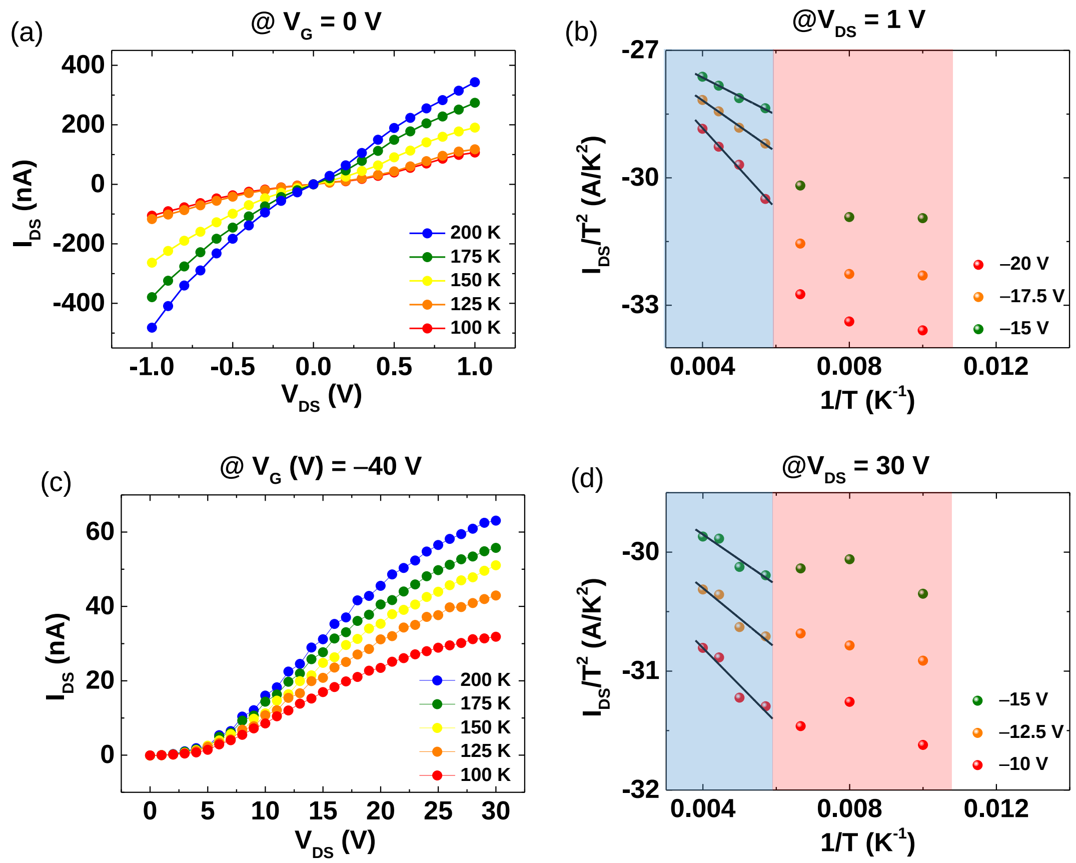

Figure S10. (a) I_{DS}–V_{DS} curves of a MoS_2 FET at various temperature from 100 K to 200 K with 25 K step. (b) Schottky barrier fitting result of MoS_2 FET from data at V_{DS} = 1 V and various gate voltages (-20, -17.5, and -15 V). Blue colored region (T > ~175 K) is well fitted with Schottky barrier model, but red colored region (T < ~175 K) is not fitted well. (c) I_{DS}–V_{DS} curves of a pentacene FET at various temperature from 100 K to 200 K with 25 K step. (d) Schottky barrier fitting result of pentacene FET from data at V_{DS} = 30 V and various gate voltages (-15, -12.5, and -10 V). Blue colored region (T > ~ 175 K) is well fitted with Schottky barrier model, but red colored region (T < ~175 K) was not fitted well.

11. Grains of pentacene on MoS_2 flake and on SiO_2

The atomic force microscopy (AFM) image of pentacene on a MoS_2 flake and a SiO_2 is shown in Fig. S11(a). And, the distribution of the grain size of pentacene on MoS_2 and SiO_2 is shown in Fig. S11(b) and S11(c), respectively. From these results, the average area of the grain size were extracted, which is summarized in Table S2. The results in Table S2 show that the grains of pentacene on MoS_2 were smaller than those on SiO_2. Smaller grain boundary could contribute to the trap site formation in the MoS_2/pentacene p-n junction system.

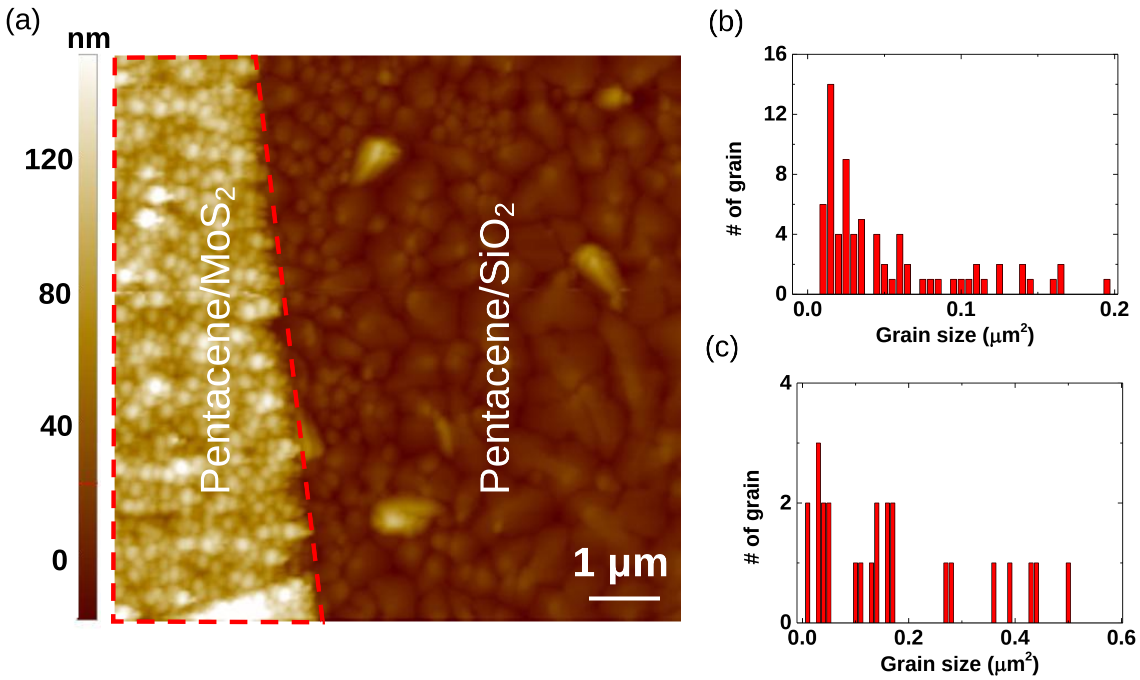

Figure S11. (a) AFM image of pentacene on a MoS_2 flake and a SiO_2. The area enclosed by red dashed box indicates pentacene layer on the MoS_2 flake. (b) Distribution of the grain size of pentacene on the MoS_2 flake and (c) on the SiO_2.

Table S2. Average grain size of the histogram of pentacene on MoS_2 and SiO_2.

5 μm × 5 μm	Average grain size (μm^2)
on MoS_2	0.086
on SiO_2	0.23

12. Cross-sectional transmission electron microscopy (TEM) images of pentacene

Figure S12 shows the cross-sectional transmission electron microscopy (TEM) images of pentacene/SiO_2 and pentacene/MoS_2/SiO_2. Uniformly deposited pentacene layer without visible defects was observed at the interface between the pentacene and SiO_2 (Fig. S12(a)). However, relatively poor uniformity of the deposited pentacene layer was observed near the MoS_2 flakes, as shown in Fig. S12(b) Defects near the MoS_2 flakes (marked as the red circles in Fig. S12(b)) can act as the interfacial trap sites.

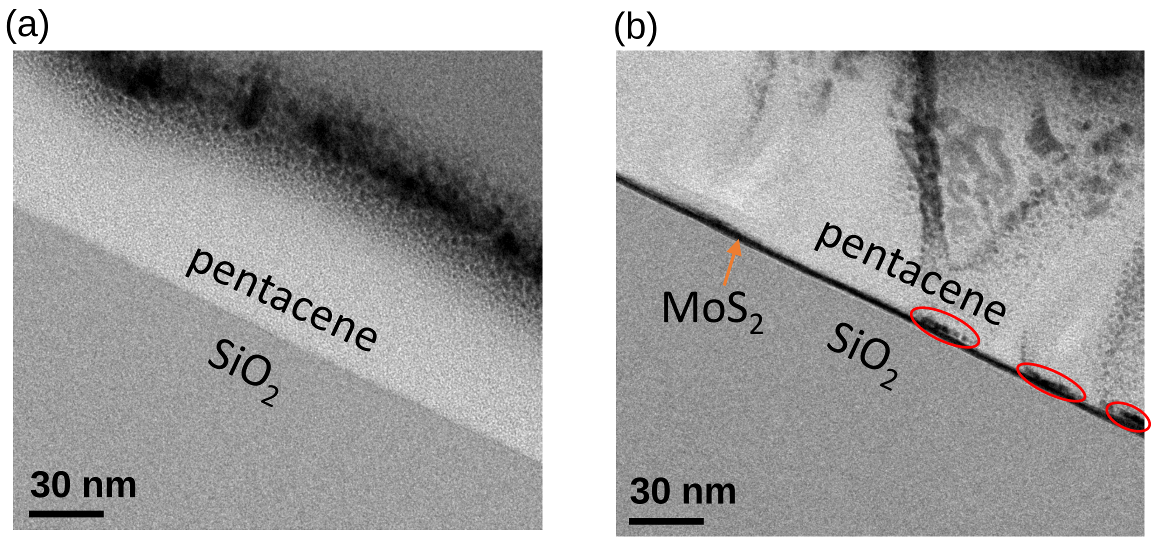

Figure S12. Cross-sectional TEM images of (a) petnacene on SiO_2 and (b) pentacene/MoS_2 p-n heterojunction.

13. AFM images of a MoS_2 flake on a SiO_2

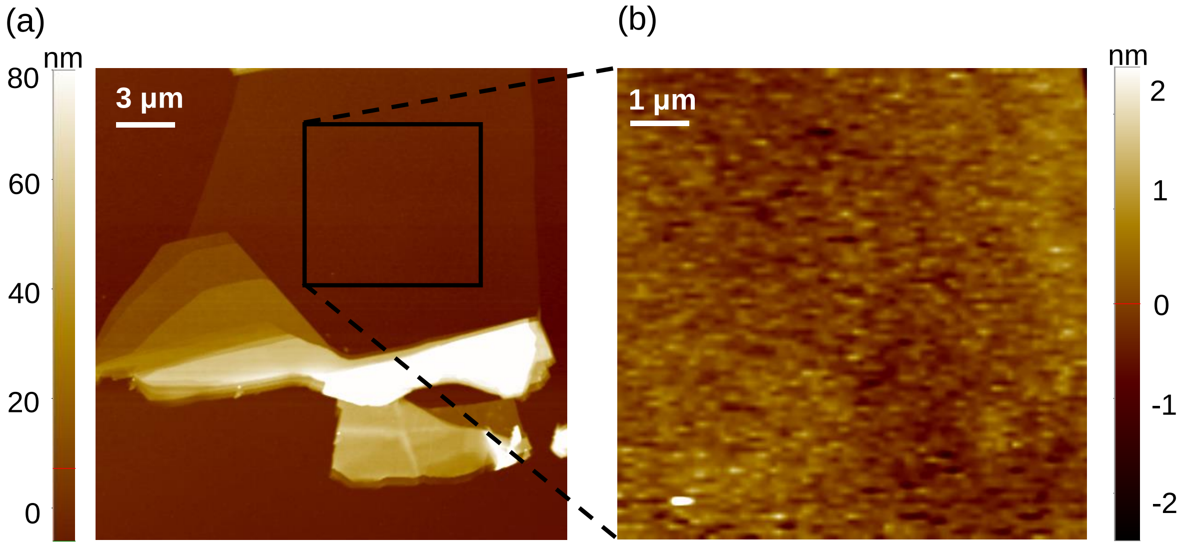

Figure S13. AFM images of (a) a MoS_2 flake and (b) a zoomed-in area.

14. AFM image of a pentacene on a SiO_2

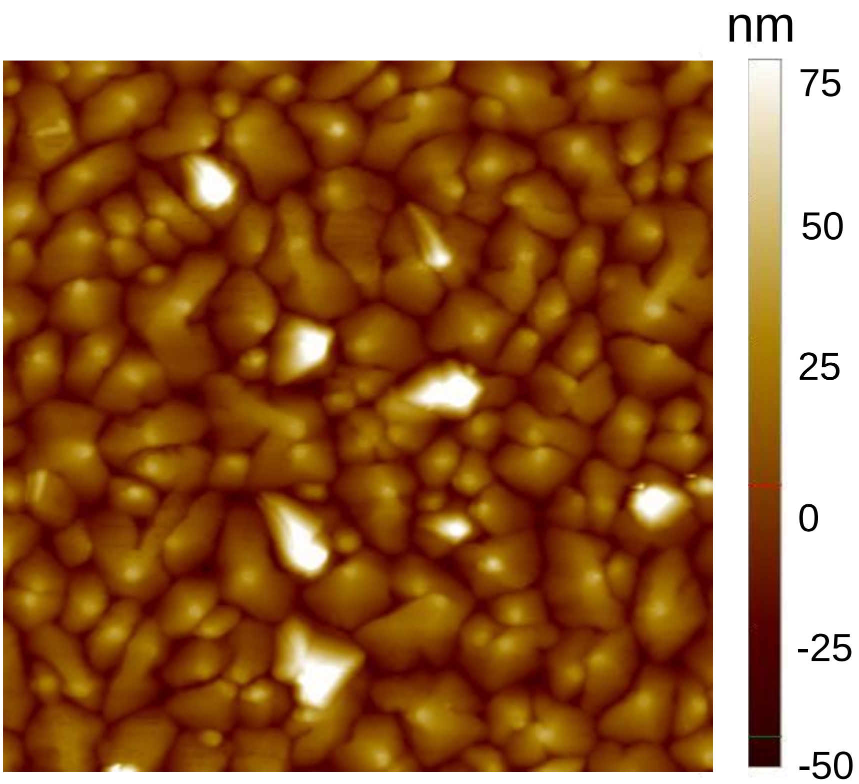

Figure S14. AFM image of a pentacene on SiO_2 substrate.

Reference

S1. Jariwala, D. et al. Band-like transport in high mobility unencapsulated single-layer MoS_2 transistors. *Appl. Phys. Lett.* **102,** 173107 (2013).

S2. Lampert, M. A. Simplified theory of space-charge-limited currents in an insulator with traps. *Phys. Rev.* **103,** 1648 (1956).

S3. Ghatak, S. & Ghosh, A. Observation of trap-assisted space charge limited conductivity in short channel MoS_2 transistor. *Appl. Phys. Lett.* **103,** 122103 (2013).
